# Roll-To-Roll Friendly Solution-Processing of Ultrathin, Sintered CdTe Nanocrystal Photovoltaics

**DOI:** 10.1021/acsami.1c08325

**Published:** 2021-09-08

**Authors:** J. Matthew Kurley, Jia-Ahn Pan, Yuanyuan Wang, Hao Zhang, Jake C. Russell, Gregory F. Pach, Bobby To, Joseph M. Luther, Dmitri V. Talapin

**Affiliations:** † Department of Chemistry and James Franck Institute, University of Chicago, Chicago, Illinois 60637, United States; ‡ Department of Electrical, Computer, and Energy Engineering, University of Colorado, Boulder, Colorado 80309, United States; § National Renewable Energy Laboratory, Golden, Colorado 80401, United States; ∥ Center for Nanoscale Materials, Argonne National Laboratory, Argonne, Illinois 60439, United States

**Keywords:** cadmium telluride, solar cell, nanocrystals, roll-to-roll, ligand chemistry, spray-coating, doctor-blading

## Abstract

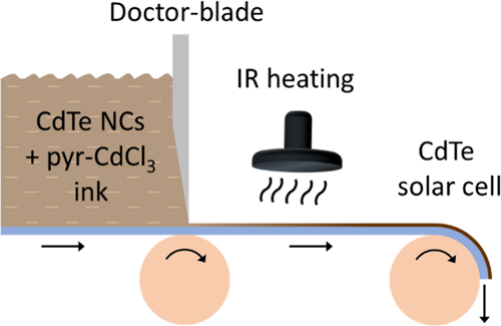

Roll-to-roll (R2R) device fabrication using solution-processed materials is a cheap and versatile approach that has attracted widespread interest over the past 2 decades. Here, we systematically introduce and investigate R2R-friendly modifications in the fabrication of ultrathin, sintered CdTe nanocrystal (NC) solar cells. These include (1) scalable deposition techniques such as spray-coating and doctor-blading, (2) a bath-free, controllable sintering of CdTe NCs by quantitative addition of a sintering agent, and (3) radiative heating with an infrared lamp. The impact of each modification on the CdTe nanostructure and solar cell performance was first independently studied and compared to the standard, non-R2R-friendly procedure involving spin-coating the NCs, soaking in a CdCl_2_ bath, and annealing on a hot plate. The R2R-friendly techniques were then combined into a single, integrated process, yielding devices that reach 10.4% power conversion efficiency with a *V*_oc_, *J*_sc_, and FF of 697 mV, 22.2 mA/cm^2^, and 67%, respectively, after current/light soaking. These advances reduce the barrier for large-scale manufacturing of solution-processed, ultralow-cost solar cells on flexible or curved substrates.

## 1 Introduction

Solution-processed solar cells assembled from roll-to-roll (R2R)-friendly techniques have garnered increasing interest over the past few decades as a low-cost alternative to single crystal silicon or chemical vapor-deposited gallium arsenide thin films.^1^ A wide variety of materials have been solution processed into photovoltaics, including organic polymers,^2−4^ lead sulfide quantum dots (PbS QDs),^5,6^ lead halide-based perovskites,^7−9^ and sintered nanocrystals^10^ (NCs) made from Cu(In,Ga)(S,Se)_2_,^11,12^ Cu_2_ZnSn(S,Se)_4_,^13−15^ or CdTe.^16,17^ Most of the top power conversion efficiencies (PCEs) are achieved using spin-coating to produce a uniform semiconductor layer. However, the inevitable disadvantages of spin-coating such as significant waste of material, modest scalability, low throughput, and planar substrate geometries severely limit the transformation of new material strategies into practically relevant technologies. Therefore, new fabrication techniques are needed to fulfill the requirements of cost reduction, speed of implementation, and flexibility. Such promising substitutes include dip-coating,^18,19^ doctor-blading,^20,21^ and spray-coating.^22,23^

Spray-coating has been proven effective at depositing PbS QDs,^24^ perovskites,^23^ and CdTe NCs.^25,26^ Foos et al. spray-coated CdTe NCs onto indium tin oxide (ITO)-coated glass and sintered the material into large grains of polycrystalline CdTe.^25^ Calcium and aluminum were thermally evaporated to create a Schottky junction solar cell, resulting in a PCE of 2.3%. Townsend et al. further improved the device PCE to 3.0% by making a heterojunction solar cell through depositing zinc oxide (ZnO) sol–gel between the CdTe absorbing layer and top electrode.^26^ Doctor-blading has also been used to deposit active layers in perovskite solar cells^20^ and in organic solar cells.^27^ CdTe NCs have been doctor-bladed on top of a vapor-phase deposited CdTe layer to reduce surface roughness and pinholes.^28^ However, there is yet to be any report on a completely R2R-friendly solution deposition of the CdTe active layer with a high PCE.

Several studies on enhancing the performance of sintered CdTe NC solar cells have been reported. Panthani et al. and MacDonald et al. improved the n-type contact to a p-CdTe absorber using In-doped sol–gel ZnO.^29,30^ They also found that current/light soaking significantly improved the contact between CdTe and ITO. However, both reports employed a saturated cadmium chloride (CdCl_2_) bath as a critical chemical treatment for achieving high PCEs. This CdCl_2_ bath step is not particularly R2R-friendly because it wastes large quantities of CdCl_2_, which is an expensive, highly toxic, and difficult-to-dispose of chemical.^31^ Additionally, in order to prevent castatrophic device failure, the bath step requires an extensive washing step to remove CdCl_2_ particulates from the substrate. By harnessing trichlorocadmate (CdCl_3_^–^) as both inorganic ligands and sintering promoters, Zhang et al. created an ink that was solution-processed and annealed into a polycrystalline CdTe absorber layer without the need for an additional chemical bath treatment.^32^ However, the NC ink lacked long-term colloidal stability and did not allow for quantitative control on the amount of sintering agent in the ink. Furthermore, virtually all the studies on sintered CdTe NCs have utilized direct conductive heating using a hotplate, which is also not R2R-friendly.

Here, we systematically explore R2R-friendly techniques for the deposition and sintering of CdTe NCs based on recent advances in NC surface ligand chemistry,^32−34^ grain growth of NC solids,^35,36^ and device interfaces.^29,30^ We first independently introduce each R2R-friendly modification to the standard procedure that involves spin-coating pyridine-capped CdTe NCs, soaking in a CdCl_2_ bath, and annealing on a hot plate. For each modification, we fabricated full device stacks so that its impact on the solar cell performance can be unambiguously determined. Spray-coating and doctor-blading techniques were demonstrated to be viable alternatives to spin-coating for the fabrication of CdTe solar cells, with only slight reductions in PCEs. These variabilities were rationalized based on the different grain morphologies produced by the different techniques. We also built upon the CdCl_3_^–^-capped CdTe NCs introduced by Zhang et al.^32^ and developed a bath-free method to quantitatively control the amount of the CdCl_3_^–^ sintering agent in our films, which allowed for controllable sintering and grain growth of the NCs. Also, infrared (IR) heating was explored as a method for efficient and R2R-friendly sintering of CdTe NCs. Finally, we showed that these modifications can be seamlessly integrated to produce a completely R2R-friendly fabrication of the CdTe layer, producing solar cells that perform comparably to those made with non-R2R-friendly techniques.

## 2 Experimental Section

### 2.1 CdTe NC Ink Preparation

#### 2.1.1 Oleate-Capped CdTe NC Synthesis

CdTe NCs capped with oleate were synthesized with a modified method described by MacDonald et al.^17,30^ In short, 4.80 g of CdO, 42.4 g of recrystallized OA, and 40.0 g of recrystallized ODE were charged in a 500 mL flask and evacuated overnight to remove trace oxygen. The flask was heated to 80 °C until the pressure equilibrated. Under dry nitrogen, the mixture was heated to 220 °C until the solution turned clear, indicating a completed reaction. The flask was cooled to <90 °C and evacuated. The flask was heated to 110° once the solution stopped bubbling and left until the pressure equilibrated. Under dry nitrogen, the flask was heated to 270 °C and 24 mL of 10 wt % TBP:Te was injected. The heating mantle was removed immediately and the flask was allowed to air cool to <50 °C. The resulting CdTe NC solution was split evenly and purified using anhydrous toluene and ethanol as the solvent/non-solvent combination.

#### 2.1.2 Pyridine Ligand Exchange and Pyridine-Capped CdTe NC Ink

Following 4–6 purification cycles, CdTe NCs were redispersed in anhydrous pyridine at a concentration of ∼80 mg/mL. The solution was stirred under N_2_ overnight on a hotplate set to 100 °C, followed by precipitation using hexane. The CdTe NC precipitates were redispersed in fresh pyridine to prepare the pyridine-capped CdTe NC stock solution. The stock pyridine-capped CdTe NC solution was precipitated by hexane and dissolved in a 1:1 mixture of pyridine and 1-PA to the desired concentration. The solution was sonicated for 10 min and filtered through a 0.2 μm polytetrafluoroethylene (PTFE) syringe filter to prepare the spin-coating solution.

#### 2.1.3 pyr-CdCl_3_ Ligand Exchange

The procedure was adapted from a process established previously by Zhang et al.^32^ In short, trichlorocadmates (CdCl_3_^–^) anions with pyridinium (pyr-H^+^) cations were synthesized by mixing equimolar amounts of CdCl_2_ and pyr·HCl in NMF (0.1 M). In a typical ligand exchange, 18 mL of oleate-capped CdTe NC (Section 2.1.1) solution in hexane (∼30 mg/mL) was mixed with 18 mL of CdCl_3_^–^ solution in NMF (0.1 M). Under vigorous stirring, NCs gradually transferred from hexane to NMF. Upon phase transfer, the bottom phase containing CdTe NCs was then rinsed with fresh hexane three times.

##### 2.1.3.1 pyr-CdCl_3_-Capped CdTe NC Ink without Proper Washing^32^

Following the CdCl_3_^–^ ligand exchange (Section 2.1.3), a mixture of toluene (6 mL) and HMPA (3 mL) was added, leading to the flocculation of NCs in solution. The NC precipitates were collected by centrifugation and re-dispersed in 5 mL of pyridine. The solution of CdCl_3_^–^-capped CdTe NCs in pyridine was vigorously stirred for ∼2 h in air, followed by centrifugation to remove the insoluble part. An equal amount of 1-PA was added to the NC solution in pyridine to make the “poorly-washed” ink.

##### 2.1.3.2 pyr-CdCl_3_-Capped CdTe NC Ink with Proper Washing Followed by the Addition of Extra pyr-CdCl_3_

Following CdCl_3_^–^ ligand exchange (Section 2.1.3), the NCs were precipitated with the same non-solvent mixture outlined previously (Section 2.1.3.1). However, instead of re-dispersing in pyridine, the NCs were dissolved in NMF (∼18 mL). The same precipitation and re-dispersing procedure were repeated. Following a third precipitation, the NCs were re-dispersed in 2.5 mL of pyridine and stirred vigorously. The solution was filtered with a 0.45 μm PTFE syringe filter to remove insoluble NCs. For simplicity, the resulting solution is referred to as the “CdTe–pyr-CdCl_3_” ink. Additional pyr-·HCdCl_3_ ligand solution in pyridine was added to the NC solution in varying amounts to replenish the Cl necessary for grain growth.

### 2.2 CdTe Absorber Layer Deposition and Treatments

#### 2.2.1 Substrate Preparation

In detail, 25 mm × 25 mm ITO-coated glass substrates (Thin Film Devices Inc.) were cleaned by sequential sonication in deionized (DI) water and Alconox detergent, DI, acetone, isopropyl alcohol (IPA), and DI. Afterward, the substrates were dried under N_2_ and hydrophilized for 10 min using a Harrick PDC-001 Extended Plasma Cleaner.

#### 2.2.2 Deposition of the CdTe NC Ink

##### 2.2.2.1 Spin-Coating

The CdTe NC inks outlined previously (Sections 2.1.2, 2.1.3.1, and 2.1.3.2) were spin-coated using the following procedure. Onto the freshly plasma-treated (Section 2.3.1) ITO substrates, the CdTe NC ink was pipetted (∼250 μL) onto the substrate and spun at 800 rpm for 30 s followed by 2000 rpm for 10 s. The substrate was transferred to a hot plate and dried at 150 °C for 2 min.

##### 2.2.2.2 Spray-Coating

The CdTe NC ink outlined previously (Sections 2.1.2, 2.1.3.1, and 2.1.3.2) was diluted with methanol by 5 parts methanol to 1 part NC solution. The layer thickness was controlled by changing the NC concentration in 1:1 pyridine/1-PA. A homemade spray-coating system was built by using a hot plate and a Paasche airbrush set to 45° to create a thinner wetting layer. The ink was loaded into the airbrush and sprayed briefly to wet the surface. The spray was controlled by solenoid valves attached to a power supply. The substrate was heated to 38 °C to facilitate drying. The spray-coating system was upgraded by making a metal turntable heated to 38 °C to move the substrates through the spray and process multiple substrates at a time. The spray nozzle was upgraded to a VMAU-316SS spraying assembly from Spraying Systems Co. to more easily adjust the spray parameters. Upon deposition, the substrate was dried at 150 °C for 2 min.

##### 2.2.2.3 Doctor-Blading

The same ink preparation procedure outlined for spray-coating (Section 2.2.2.2) was used. An Al block was heated on a hot plate to 40 °C to facilitate smooth deposition. Glass slides were placed on the block to act as height guides. A small amount of the NC solution (∼75 μL) was pipetted onto the substrate and a glass rod was used to smooth the film by moving back and forth. The excess was wicked away by sweeping the rod onto the glass slides. Upon deposition, the substrate was dried at 150 °C for 2 min.

#### 2.2.3 Chemical and Thermal Treatment

##### 2.2.3.1 CdCl_2_ Bath and Annealing for CdTe/Pyridine

For the CdCl_2_ treatment, the substrate was cooled in air and was dipped into a saturated CdCl_2_ bath in methanol at ∼60 °C for 15 s, thoroughly rinsed with IPA, and dried under a N_2_ flow. The substrate was annealed at 350 °C on a hot plate (or under an IR lamp shielded with Al foil) for 20 s and cooled in air. The whole process (deposition, drying, CdCl_2_ treatment, thermal treatment) was repeated multiple times (12–20) until the desired thickness was achieved.

##### 2.2.3.2 Annealing Only

For CdCl_3_^–^-capped CdTe NC inks, there was no need for a CdCl_2_ bath treatment. Instead, the substrate was transferred directly from the drying plate to the annealing plate. The substrate was annealed at 350 °C on a hot plate (or under an IR lamp shielded with Al foil) for 20 s and cooled in air. The whole process (deposition, drying, annealing) was repeated multiple times (12–20) until the desired thickness was achieved.

#### 2.2.4 Spray-Coating onto Curved Substrates

Glass rods, beads, and plano-convex lenses were purchased from various outside vendors. They were affixed to the spray-coater using double-sided tape. For full devices, special holders would be necessary to assure consistency and reduce mistakes from processing difficulties.

### 2.3 Finishing CdTe Solar Cells

#### 2.3.1 ZnO n-Type Layer

The ZnO layer was deposited on top of CdTe by spin-coating 300 μL of the ZnO sol–gel at 3000 rpm for 30 s, followed by annealing at 300 °C for 2 min. The ZnO sol–gel was prepared by sonicating a mixture of 1.50 g of Zn(OAc)_2_·2H_2_O, 15 mL of 2-methoxyethanol, 420 μL of ethanolamine, and 15–45 mg of InCl_3_ for 1 h and subsequently stirring overnight.

#### 2.3.2 Electrode Deposition

The substrates were transferred into a glovebox and kept under high vacuum (∼10^–9^ Torr) overnight. Top Al contacts (100 nm) were deposited by thermal evaporation through a homemade mask, featured by evenly distributed 8 mm^2^ holes. Ag (100 nm) was deposited on top of Al to increase device longevity. Three sides of the device stack were scratched off to expose the ITO. Electrical contact was established using Ag paint.

### 2.4 Characterization Techniques

The optical absorption spectra of NC solutions were collected using a Cary 5000 UV–vis–NIR spectrophotometer in transmission mode. Scanning electron microscopy (SEM) images of the complete CdTe solar cell devices were acquired on a Zeiss-Merlin instrument. X-ray photoelectron spectroscopy (XPS) analysis was performed on a Kratos AXIS Nova spectrometer using a monochromatic Al Kα source (*h*ν = 1486.6 eV). The Al anode was powered at 10 kV and 15 mA. Instrument base pressure was 1 × 10^–9^ Torr. High-resolution spectra in Cd 3d, Te 3d, C 1s, Cl 2p, and P 2p regions were collected using an analysis area of 0.3 × 0.7 mm^2^ and a 20 eV pass energy. Wide-angle powder X-ray diffraction (XRD) patterns were collected using a Bruker D8 diffractometer with a Cu Kα X-ray source operating at 40 kV and 40 mA.

### 2.5 Photovoltaic Characterization

Devices were tested under the illumination of a Xe lamp with a AM 1.5G filter (Newport 67005) and calibrated with a Si photodiode with a KG5 filter (Hamamatsu Inc, S1787-04). The illumination area was controlled by a self-aligning stainless-steel aperture mask with evenly distributed, nominal 6 mm^2^ circular holes (5.94 mm^2^ measured). Current density versus voltage (*JV*) curves were acquired using a Keithley 2400 SourceMeter controlled by a LabVIEW interface. To mitigate heating during measurements, the perimeter of the cell was in direct contact to an Al heat sink. The instruments were controlled and data were collected using a homemade LabVIEW program. Current/light soaking was done by applying 2–3 V (forward bias) to the device under illumination for varying amounts of time. Typically, this generated a current density of ∼2.5 A cm^–2^. The current was monitored carefully to not exceed 3 A cm^–2^ as current densities greater than this generally caused performance degradation. Holding the devices in reverse bias generally caused a transient decrease in performance (due to reduced *V*_OC_). External quantum efficiency (EQE) measurements were taken using an Oriel IQE-200 with a step of 20 nm for the wavelength. Capacitance–voltage (Mott–Schottky) data were acquired using a Gamry Reference 600 potentiostat. Data were acquired using a frequency of 500 Hz with an amplitude and step size of 5 and 10 mV, respectively.

## 3 Results and Discussion

### 3.1 Evaluation of Scalable Deposition Techniques

Spin-coating proves difficult to integrate into a R2R process. It also wastes considerable amounts of material, requires batch processing, and limits the geometry to planar substrates. All these factors make other deposition methods, such as doctor-blading or spray-coating, better alternatives.

Using a homebuilt spray-coating system (Figure S1), we tested the spray-coating deposition method by starting with pyridine-capped CdTe NCs previously described by Jasieniak et al.^17^ and Panthani et al.^29^ Initially, we deposited films using the ink containing 40 mg/mL CdTe NCs in a 50/50 mixture of pyridine and 1-propanol as previously reported. The resulting films were uneven, calling for an alternative solvent combination. We tried a variety of solvents (chloroform, pyridine, or methanol) to better facilitate deposition. Eventually, diluting by 6 times with methanol (6 mg/mL in a 1:1:5 ratio of pyridine/1-propanol/methanol) proved successful at improving the film quality (Figure S2). Methanol increases surface wetting and evaporates quickly enough to leave a thin, wetted layer of NC solution on the substrate. This allows the film to solvent anneal to remove defects, improving the uniformity.^37^ This solvent mixture was also suitable for doctor-blading, yielding films with good smoothness and uniformity (Figure S3).

**Figure 1:**
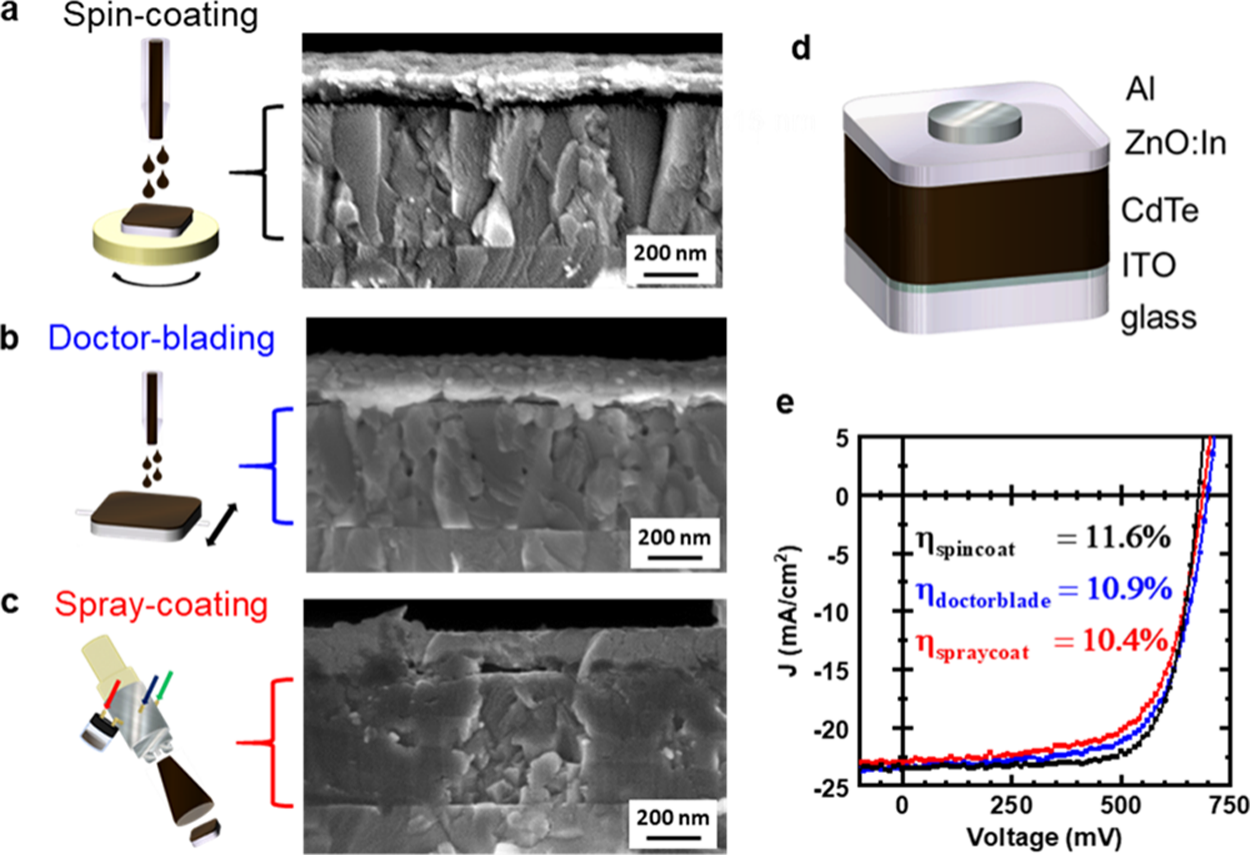
(a–c) Cross-sectional SEM images of solar cell devices made by spin-coating (a), doctor-blading (b), or spray-coating (c) the CdTe NC layer, showing the differences in grain morphology. The CdTe layer was deposited via a layer-by-layer method using pyridine-capped CdTe NCs, soaking in a CdCl_2_ bath, and annealing on a hot plate. (d) Schematic of the standardized device architecture (ITO/CdTe/ZnO:In/Al). (e) *J*–*V* curves and PCEs for devices fabricated by spin-coating (black), doctor-blading (blue), or spray-coating (red) after current/light soaking.

The optimized CdTe ink was then used to spray-coat or doctor-blade an ∼500 nm thick CdTe layer via a layer-by-layer method (with the use of a CdCl_2_ bath and hot plate annealing), which we compared to CdTe deposited by spin-coating (Figure 1). Cross-sectional SEM revealed that the morphology of the grains was strongly dependent on the deposition method. Spin-coating yielded columnar-like grains, with many grains spanning continuously across the entire layer thickness (Figures 1a and S4). This anisotropic grain growth suggests enhanced recrystallization of NCs on the exposed side of the existing grains during the layer-by-layer assembly. Doctor-bladed films had similar columnar-like structures but were more discontinuous and disordered on the nanoscale (Figures 1b and S4). Furthermore, they contained 10–50 nm sized holes consistently found throughout the entire layer. We attribute this to lower nanoscale uniformity of the doctor-bladed layers (compared to spin-coated layers) due to a longer drying time, similar to previous observations in the deposition of polymer photovoltaic layers.^38^ Spray-coating resulted in CdTe layers that were significantly different compared to the other two methods (Figures 1c and S4). The grains appeared to be more isotropic and varied more in size. The presence of wide grains indicates that there was more grain growth in the lateral dimension. These effects could be attributed to the deposition of NCs normal to the surface during spray-coating, which helps fill up the crevices and promote lateral grain growth. This contrasts with spin-coating and doctor-blading, which utilizes forces parallel to the surface for deposition. However, the spray-coated layers have a larger micro-scale variability in the layer thickness, which stems from less controlled deposition uniformity compared to the other two techniques.

We compared the solar cell performance of the three deposition techniques using a previously used, simple device architecture (Figure 1d).^29,32^ Although this device architecture is not optimal for practical implementation due to the energetic mismatch at the CdTe/ITO interface, it allows the light-harvesting quality of the CdTe layer to be compared. For each technique, we tested nine devices (on a single substrate) and obtained their device statistics (Figure S10). The analysis shows that the three techniques produced solar cells with similar short circuit current density, *J*_SC_, while doctor-bladed devices had a slightly higher open-circuit voltage, *V*_OC_, compared to the other two methods. This resulted in a higher average PCE for the doctor-bladed devices.

To show the true potential of the devices, we carried out a current/light soaking step (which reduces the energetic misalignment at the CdTe/ITO interface) on the best device from each substrate. Upon current/light soaking, the PCE of the spin-coated devices is the best, followed by doctor-bladed devices (Figure 1e). Interestingly, the *V*_OC_ and *J*_SC_ of all three deposition methods are now virtually equivalent within the reasonable variability in device thicknesses and processing conditions. Instead, the lower efficiencies of the spray-coated and doctor-bladed devices can be attributed to their smaller fill factors. Further analysis (Figure S5 and Table 1) shows that this can be traced to both larger series resistances and smaller shunt resistances of these devices when compared with spin-coated devices. The larger series resistance is likely from the increase in grain boundaries due to the presence of smaller grains. The shunt resistance of the doctor-bladed devices is particularly low, which is consistent with the pinholes present in its SEM images. Nonetheless, PCEs in excess of 10% are achievable for these more scalable deposition techniques that were implemented with relatively simple home-built apparatuses.

**Table 1:** Figures of Merit of the Best Solar Cells Made by Depositing the CdTe Layer by Different Techniques

deposition	*V*_OC_ (V)	*J*_SC_ (mA/cm^2^)	PCE (%)	fill factor (%)	*R*_series_ (Ω cm^2^)	*R*_shunt_ (Ω cm^2^)
spin-coating	0.676	23.4	11.6	73.2	2.4	14.4 × 10^2^
doctor-blading	0.697	23.4	10.9	66.8	3.5	3.8 × 10^2^
spray-coating	0.686	23.0	10.4	65.9	3.6	6.3 × 10^2^

The material efficiencies for the various deposition techniques were also calculated. Spin-coating is well known to be an inefficient method for depositing any ink. For our system specifically, 200 μL of 40 mg/mL NC ink was required to cover a single 25 mm × 25 mm substrate. Over the course of 20 layers, ∼500 nm of CdTe is deposited onto the substrate. Therefore, ∼160 mg of NC is required to deposit ∼1.9 mg of CdTe. The result is only ∼1% of material remains on the substrate during spin-coating. For the same thickness, 80 mg of CdTe NC was required during spray-coating (∼2% material efficiency), and only 2 mg was required for doctor-blading (∼95% material efficiency). Note that the material efficiency for spray-coating should increase significantly for larger areas, with the literature reporting spray-coating material efficiencies as high as ∼95% by Gilmore et al.^39^

**Figure 2:**
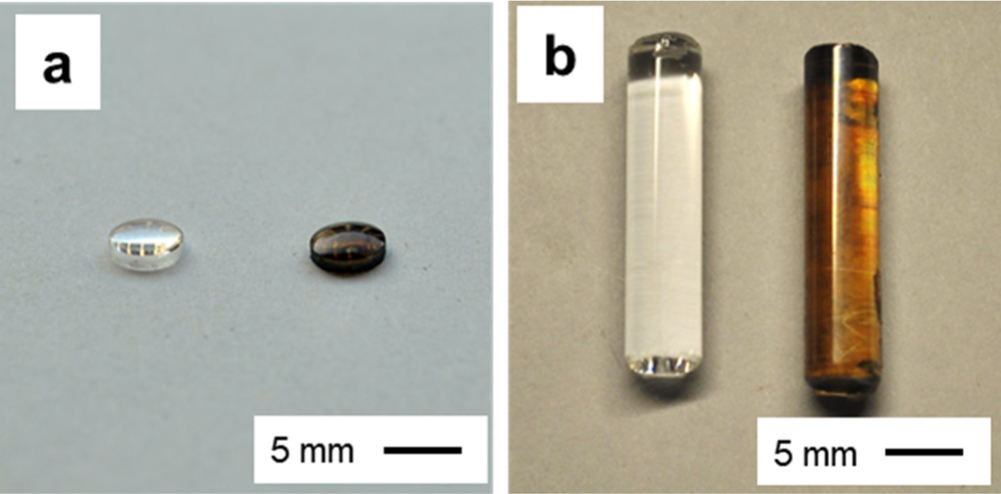
(a,b) Images of plano-convex lenses (a) and cylindrical rods (b) that were freshly cleaned (left) and spray-coated with CdTe NC ink (right).

To further demonstrate the versatility of spray-coating, we sprayed a smooth layer of NCs onto a variety of curved substrates, including plano-convex lenses and cylindrical rods (Figure 2). This flexibility can enable the deposition of solar cells on substrate geometries that concentrate/direct light and potentially even on everyday objects (*e.g.* cars, buildings).

### 3.2 Bath-Free, Controllable Sintering of CdTe NCs

**Figure 3:**
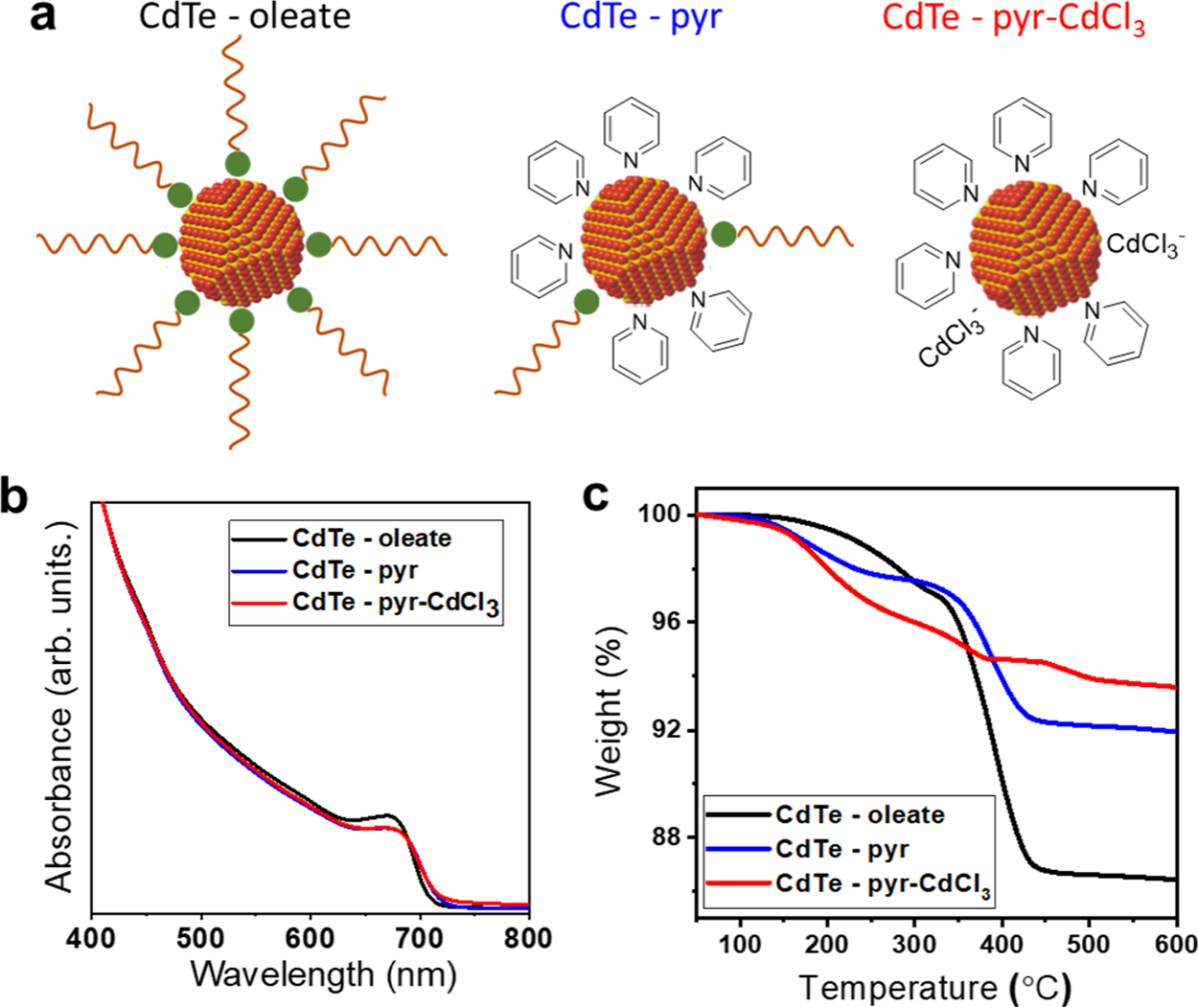
(a) Schematic of CdTe NCs capped with oleate (left) after simple pyridine exchange (middle) and after pyr-CdCl_3_ treatment (right). (b,c) Normalized absorption spectra (b) and TGA (c) of CdTe NCs before and after the different ligand exchanges.

In the standard CdTe NC processing method that uses a CdCl_2_ bath, the oleate-capped NCs (Figure 3a, left) are first exchanged with pyridine by heating the NCs in excess pyridine. Pyridine acts both as a solvent and as an L-type ligand which removes some of the oleate ligands in the form of pyridine-Cd-(oleate)_2_, resulting in pyridine-capped CdTe NCs (Figure 3a, middle).^40^ Although this results in well-dispersed NCs (Figure 3b), thermogravimetric analysis (TGA) indicates the presence of residual oleate ligands by the significant weight loss starting around 350 °C (Figure 3c), which agrees with previous findings.^40,41^ The residual oleate ligands, however, can be removed during the CdCl_2_ bath step, allowing the fabrication of highly efficient solar cells.^17^

We have previously shown that this non-R2R-friendly CdCl_2_ bath step could not be replaced by simply adding CdCl_2_ to pyridine-capped CdTe NCs.^32^ This has been attributed to the presence of residual insulating oleate, which leads to extremely poor solar cell performance. Instead, a two-phase exchange with pyridinium trichlorocadmate (pyr-CdCl_3_) was critical in fabricating high-performing solar cells without the use of the CdCl_2_ bath step. However, in order to preserve the amount of CdCl_2_, the NC ink was not thoroughly washed from unbound ligands, which caused it to lack long-term colloidal stability.

Here, we modified this method by using the same two-phase ligand exchange with pyr-CdCl_3_ as demonstrated previously but with three rounds of washing to remove excess pyr-CdCl_3_. This process yielded stabilized pyr-CdCl_3_-capped CdTe NCs (Figure 3a, right) without any pronounced NC etching (Figure 3b). FTIR analysis shows the removal of most of the oleate ligands (Figure S6), while TGA shows a smaller weight loss upon annealing to 600 °C compared to pyridine-capped NCs (Figure 3c). Instead of oleate, we attribute this small weight loss to the decomposition of small amounts of bound pyr-CdCl_3_. This is further corroborated by the small but unmistakable detection of the Cl 2p signal using XPS (Figure S7b, red).

**Figure 4:**
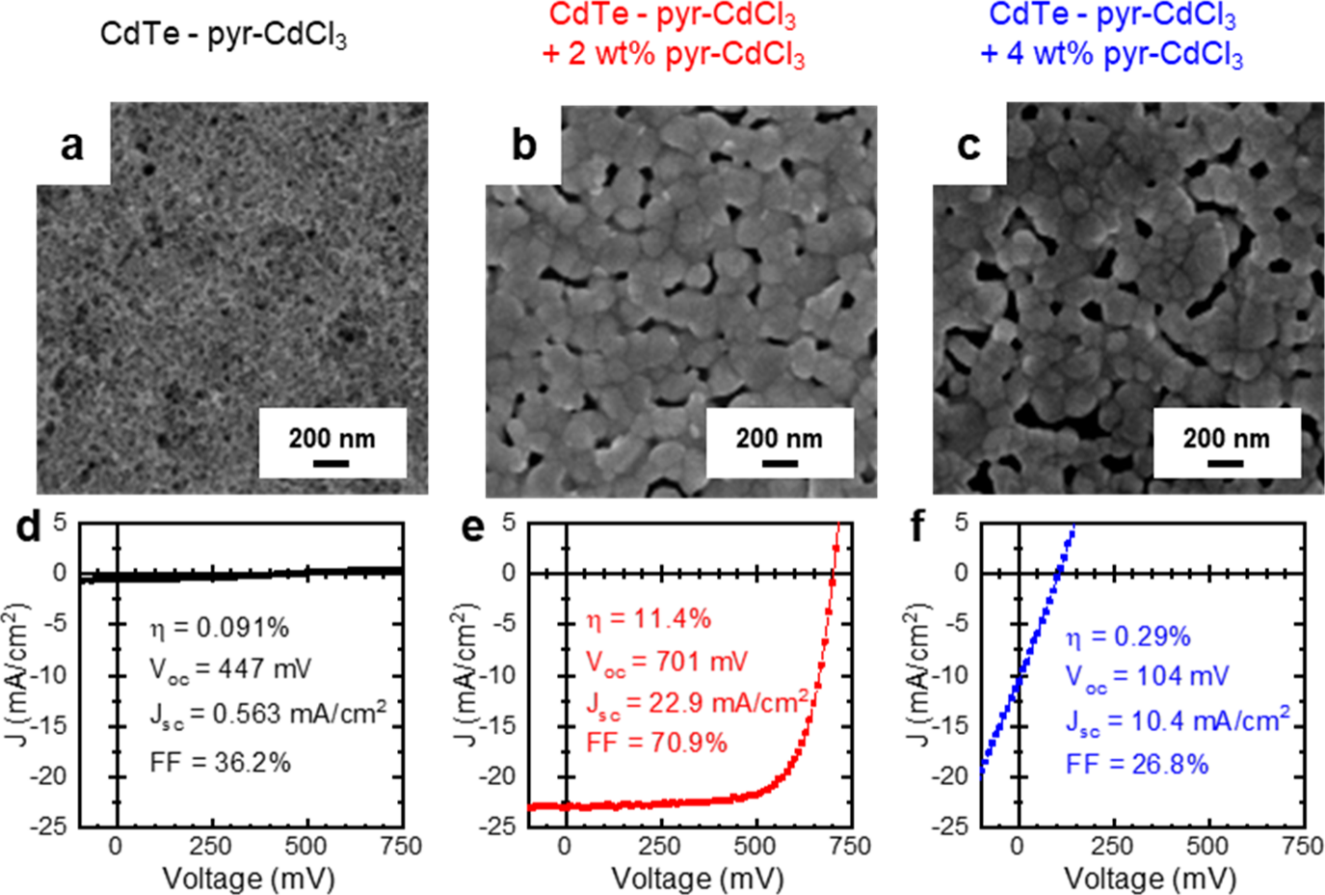
(a–c) Top-view SEM images of a single-layer CdTe thin film annealed at 350 °C for 20 s in air made from pyr-CdCl_3_-capped CdTe NCs with the addition of 0 (a), 2 (b), and 4 wt % (c) pyr-CdCl_3_. (d–f) *JV* curves from devices made with pyr-CdCl_3_-capped CdTe NCs with the addition of 0 (d), 2 (e), and 4 wt % (f) pyr-CdCl_3_.

The amount of pyr-CdCl_3_ bound to the NCs after proper washing is not enough to induce grain growth of the NCs upon annealing at 350 °C as shown by SEM images (Figure 4a) and XRD patterns (Figure S7e). As a result of the small grain sizes and significant Te oxidation observed by XPS (Figure S7c, red), devices made from these NCs exhibited a drastic decrease in short-circuit current density (*J*_SC_) and a negligible PCE due to its large series resistance of ∼500 Ω cm^2^ (Figures 4d and S11). To induce grain growth, an additional pyr-CdCl_3_ ligand can be added to the NC ink. Since this addition slowly de-stabilizes the NCs (over several hours), this step was only carried out immediately prior to deposition. Hence, the well-stabilized pyr-CdCl_3_-capped CdTe NC ink can be kept for a longer period compared to the previously poorly washed inks that destabilize within a few hours. This subtle but crucial modification is important for larger-scale processing that involves a longer time delay between ink formulation and deposition.

We added an additional 2 or 4 wt % pyr-CdCl_3_ (with respect to the solid CdTe NC weight) to the ink and investigated its effect on grain growth and solar cell performance. The addition of 2 wt % ligand led to a significant growth of the CdTe grains when annealed at 350 °C (Figures 4b and S7e) and the reduction of Te oxidation (Figure S7c,d green). Standard devices using this ink reached a maximum PCE of 11.4% upon current/light soaking with the open-circuit voltage (*V*_oc_), *J*_sc_, and fill factor (FF) reaching 701 mV, 22.9 mA/cm^2^, and 71%, respectively (Figure 4e). Device statistics before current/light soaking is shown in Figure S10. Increasing the amount of pyr-CdCl_3_ added to 4 wt % resulted in similar grain sizes (Figures 4c and S7e) and even less Te oxidation (Figure S7c,d, blue). However, it also produces more pinholes in the film, which leads to catastrophic device shorting as shown by its low shunt resistance (Figures 4f and S11), which prevents effective light harvesting. In summary, we established a bath-free technique allowing the incorporation of appropriate amounts of the sintering agent for good CdTe solar cell performance.

### 3.3 IR Heating

The annealing step is important in promoting grain growth of the CdTe NCs, and its parameters have been thoroughly optimized. The use of hot plates constrains device fabrication to batch-by-batch processing, which motivated us to use IR heating to more accurately simulate conditions in R2R-fabrication. IR lamps are R2R-friendly since they deliver radiative heat, providing zonal heating as opposed to surface heating associated with hot plates. Previous reports determined optimal conditions for annealing were 350 °C for 20 s.^17^ For consistency, we attempted to keep the same conditions. To achieve the desired temperature using an IR lamp, we found it necessary to create an isolated atmosphere protected from air flow to prevent heat loss. With this IR heating system, we fabricated devices that achieved comparable solar cell efficiencies to those made by hot plate annealing (Figure S8).

### 3.4 Integration of R2R-Friendly Techniques

**Figure 5:**
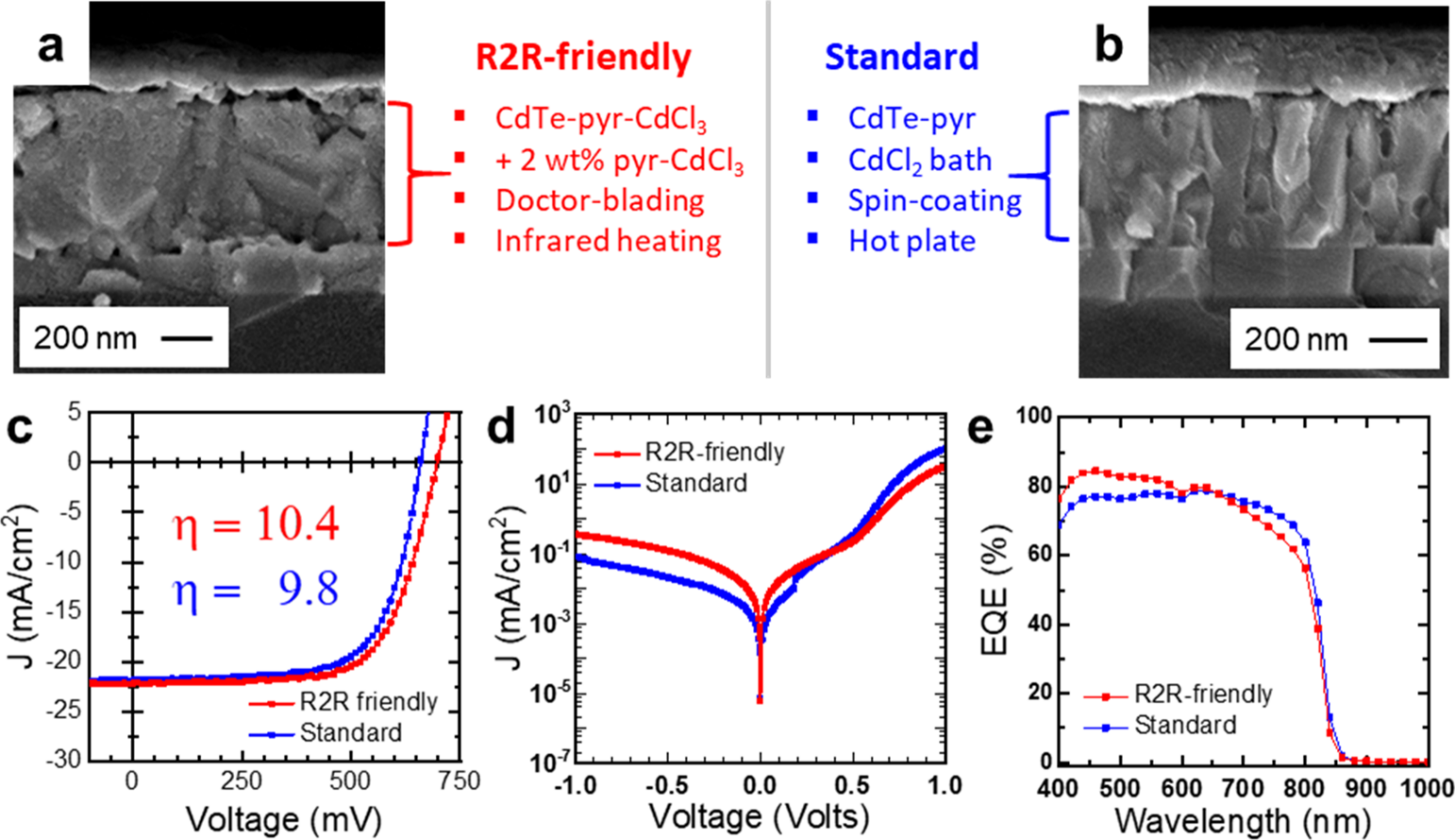
(a,b) Cross-sectional SEM of devices made with R2R-friendly techniques (a) or with a standard, non-R2R-friendly method (b) using the same batch of CdTe NCs. (c–e) *JV* curves under AM 1.5G illumination (c), dark *JV* curves (d), and EQE (e) for the best devices made by both techniques after current-light soaking.

To show that our various R2R-friendly techniques can be integrated seamlessly, we made devices that utilize all these modifications together and compared them to devices made with the standard, non-R2R-friendly techniques (Figure 5). The R2R-friendly approach utilizes pyr-CdCl_3_-capped CdTe NCs with an additional 2 wt % pyr-CdCl_3_ added immediately prior to usage. This ink was deposited with doctor-blading and annealing with an IR lamp. We also did a side-by-side comparison by fabricating devices with the standard technique that involves spin-coating pyridine-capped CdTe NCs from the same synthesis batch, soaking in a CdCl_2_ bath, and annealing with a hot plate.

Cross-sectional SEM images (Figures 5a,b and S9) showed that the R2R-friendly deposition yielded larger and more continuous grains, particularly in the lateral direction. Interestingly, the issue of small 10–50 nm holes present in doctor-bladed films (Figure 1b) was not present in these films. Overall, the R2R-friendly approach was found to have a very similar solar cell performance when compared side-by-side to the standard approach (Figure 5c–e). The best PCE achieved with the R2R-friendly approach after current/light soaking was 10.4%, with *V*_oc_, *J*_sc_, and FF being 697 mV, 22.2 mA/cm^2^, and 67%, respectively. Device statistics before current/light soaking (Figure S10) show a comparable variance in the figures-of-merits. Compared to the standard approach, the R2R-friendly method has a slightly steeper drop in EQE at longer wavelengths. This could be due to a higher recombination rate near the back CdTe/ZnO interface due to higher surface roughness.

## 4 Conclusions

We developed a procedure that integrates doctor-blading and spray-coating, CdCl_3_^–^ surface ligands, and IR-assisted heating into an R2R-friendly process (Scheme S1). Transitioning to spray-coating or doctor-blading allows for a continuous process stream without the need to load the substrate onto a vacuum chuck. Furthermore, these deposition techniques have a profound effect on grain growth, making it an important parameter for the fabrication of more efficient devices. CdCl_3_^–^ surface chemistry eliminates the need for the CdCl_2_ bath treatment, decreasing the overall number of steps and Cd-containing waste. Instead, the ink is self-contained, comprising the CdTe NCs and the pyr-CdCl_3_ grain growth promoter. Moreover, the pyr-CdCl_3_ can be added in controllably, allowing the further tuning of NC sintering and device performance. Changing from a hot plate to an IR lamp proves that the substrate does not need to be heated from the glass side to create continuous grains of CdTe throughout the film, further increasing the viability of R2R processing. By integrating these three R2R-friendly modifications in a high-PCE device, we show the viability of combining fundamental chemistry principles and applied engineering optimization to understand and develop cheap and efficient photovoltaic devices utilizing colloidal NCs.

## Supporting Information Available

Chemical information, picture of a spray-coater, pictures of spray-coated films with various solvent combinations, pictures comparing CdTe layers deposited by spin-coating and doctor-blading, additional SEM images, fittings to obtain shunt and series resistance, FTIR analysis of the CdTe ink, XPS spectra, *JV* curves of solar cells made with IR lamp heating, scheme of the integrated R2R-friendly deposition, and statistics of device performance before current/light soaking (PDF)
