# Supplementary material for: Roll-To-Roll Friendly Solution-Processing of Ultrathin, Sintered CdTe Nanocrystal Photovoltaics
Source: ACS Appl Mater Interfaces. 2021 Sep 8;13(37):44165–73. doi: 10.1021/acsami.1c08325 (PMC8461606; doi:10.1021/acsami.1c08325)
Supplement: Supplementary file 1 — am1c08325_si_001.pdf [file am1c08325_si_001.pdf]

Supporting Information

Roll-to-Roll Friendly Solution-Processing of Ultrathin, Sintered CdTe Nanocrystal Photovoltaics

J. Matthew Kurley,[†,§] *Jia-Ahn Pan,*[†,§] *Yuanyuan Wang,*[†] *Hao Zhang,*[†] *Jake C. Russell,*[†] *Gregory Pach,*[‖,°] *Bobby To,*[°] *Joseph M. Luther,*[°] *and Dmitri V. Talapin*[*,†,‡]

† Department of Chemistry and James Franck Institute, University of Chicago, Chicago, Illinois 60637, USA

‖ Department of Electrical, Computer, and Energy Engineering, University of Colorado, Boulder, Colorado 80309, United States

° National Renewable Energy Laboratory, Golden CO, 80401 USA

‡ Center for Nanoscale Materials, Argonne National Laboratory, Argonne, Illinois 60439, USA

§ These authors contributed equally

Corresponding Author

* E-mail: dvtalapin@uchicago.edu

1. Chemicals. Cadmium oxide (CdO, 99.99+%), tellurium shot (Te, 99.999%), cadmium chloride ($CdCl_2$, 99.99%), pyridinium hydrochloride (pyr·HCl, 98%), zinc acetate dihydrate ($Zn(OAc)_2 \cdot 2H_2O$, 99.999%), indium chloride ($InCl_3$, 99.999%), oleic acid (OA, technical grade, 90%), 1-octadecene (ODE, technical grade, 90%), tributylphosphine (TBP, 97% with isomers), toluene (≥99.8%, anhydrous), ethanol (≥99.5%, anhydrous), pyridine (99.8%, anhydrous), hexane (95%, anhydrous), *N*-methylformamide (NMF, 99%), hexamethylphosphoramide (HMPA, 99%), 1-propanol (1-PA, 99.7%, anhydrous), 2-methoxyethanol (99.9%, anhydrous), and ethanolamine (99.5%, redistilled) were purchased from Aldrich. Molecular sieves grade 564 (Type 3A, 8-12 mesh beads), acetone (certified ACS), methanol (certified ACS), and 2-propanol (IPA, certified ACS) were purchased from Fisher Scientific. Aluminum (Al, 99.99%) and silver (Ag, 99.99%) pellets were purchased from Kurt J. Lesker Company. PELCO colloidal silver paste (Ag paste) was purchased from Ted Pella, Inc. 10 wt% TBP:Te was prepared by dissolving 10 g of Te shot in 90 g of TBP overnight in a N_2-filled glove box. ODE was recrystallized by cooling the bottle in a chiller overnight at 12 °C and decanted to remove impurities. OA was cooled to 16 °C overnight and vacuum filtered to remove high melting point impurities. Pyridine, NMF, and 1-PA were distilled to remove low and high boiling point impurities. NMF and HMPA were dried over molecular sieves and filtered with 0.2 μm PTFE filter before use.

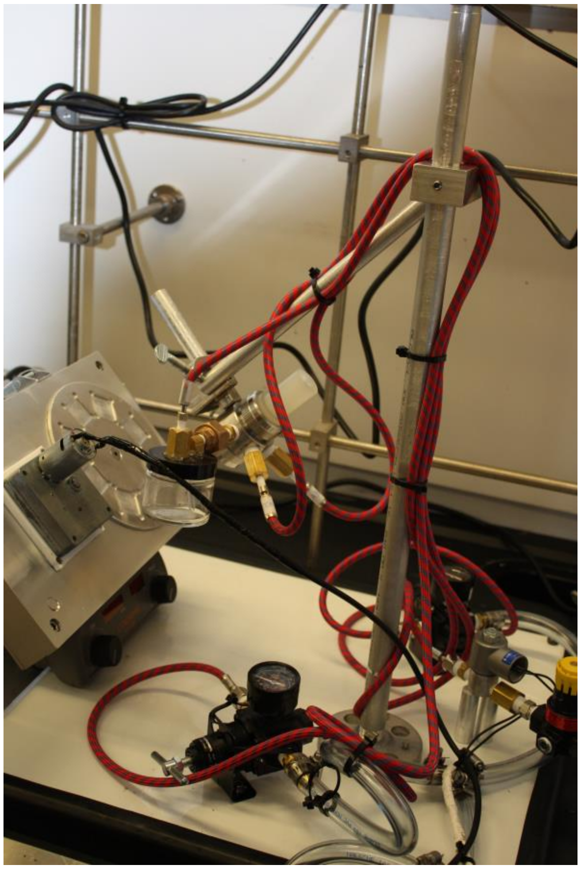

Figure S1. Picture of the home-built spray-coating system.

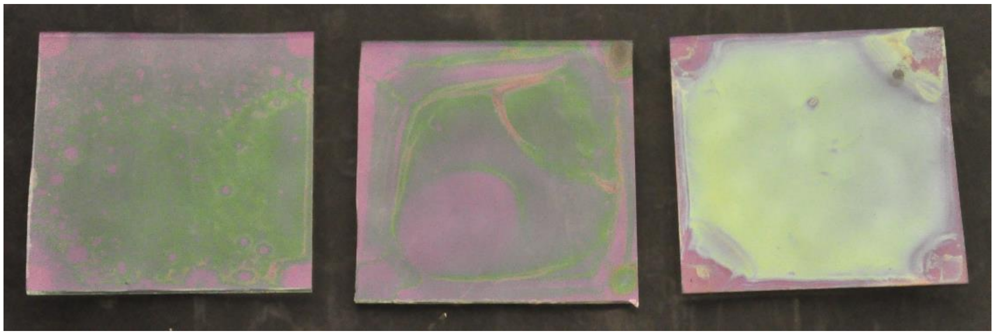

Figure S2. Substrates spray-coated using 60 mg/mL pyridine-capped CdTe NCs in a 50/50 mixture of pyridine and 1-propanol as a base. The solution was diluted to 6 mg/mL in chloroform (left), 50/50 pyridine/1-propanol (middle), and methanol (right).

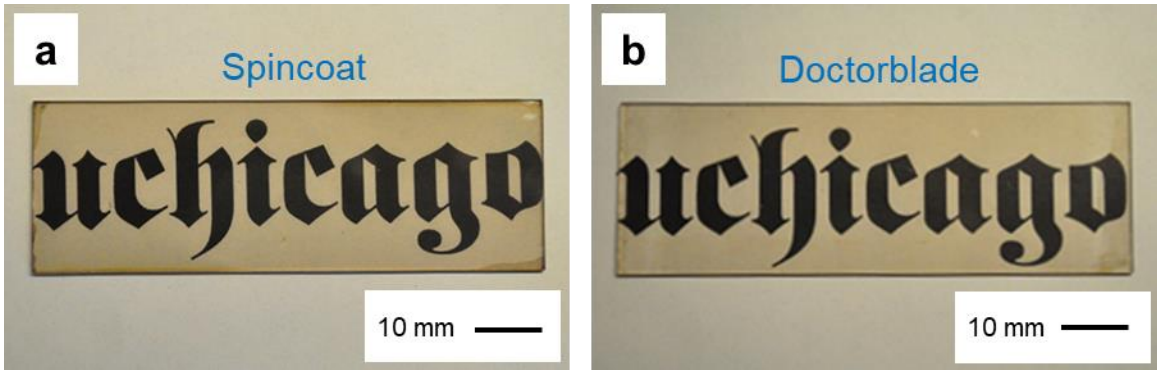

Figure S3. A single layer of CdTe NCs deposited on a glass slide via spin-coating (a) or doctor-blading (b). The good uniformity of the film is evident from the clarity of the printed "uchicago" characters on a paper placed behind

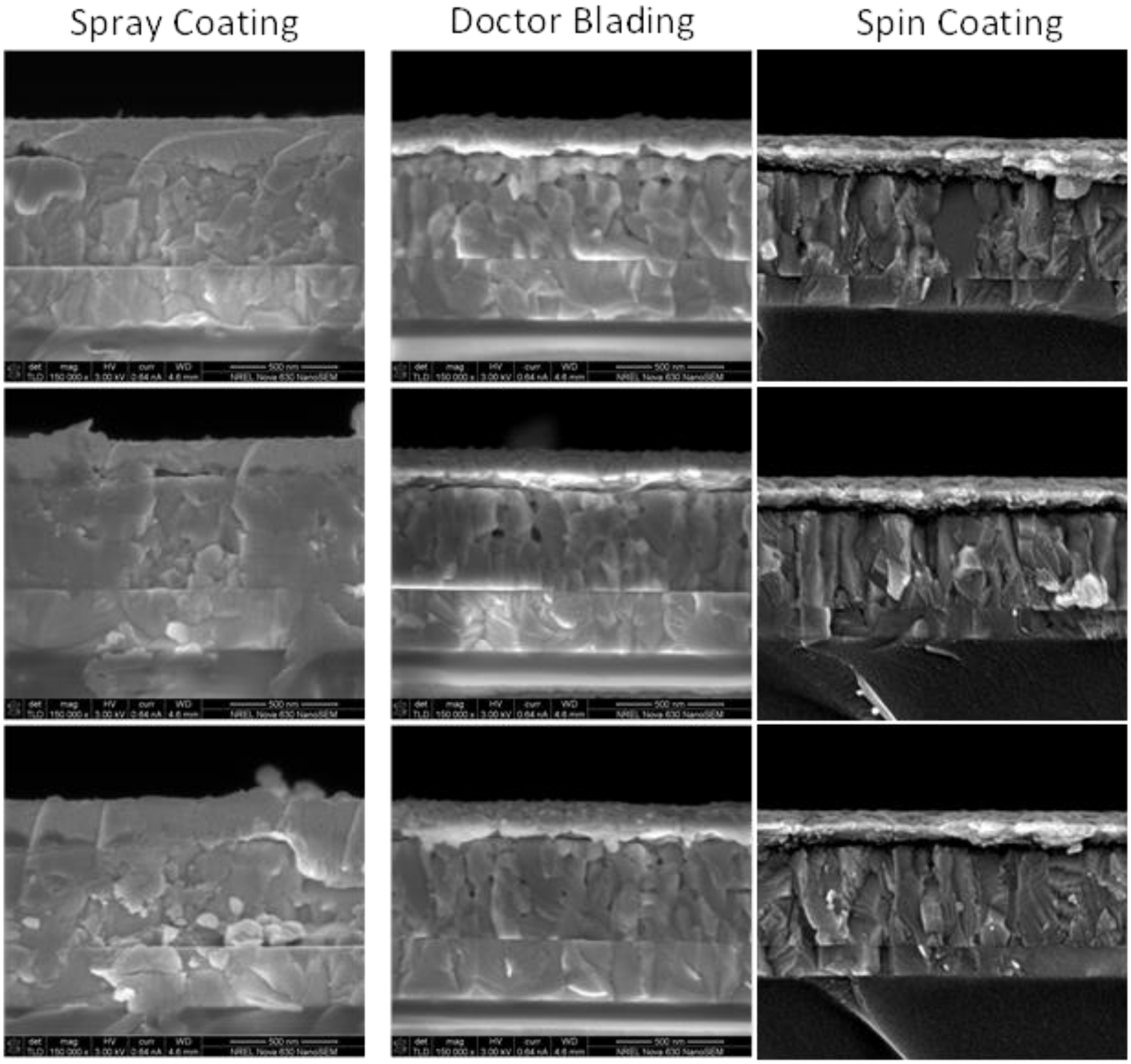

Figure S4. Additional cross-sectional SEM images of spin-coated, spray-coated and doctor-bladed devices.

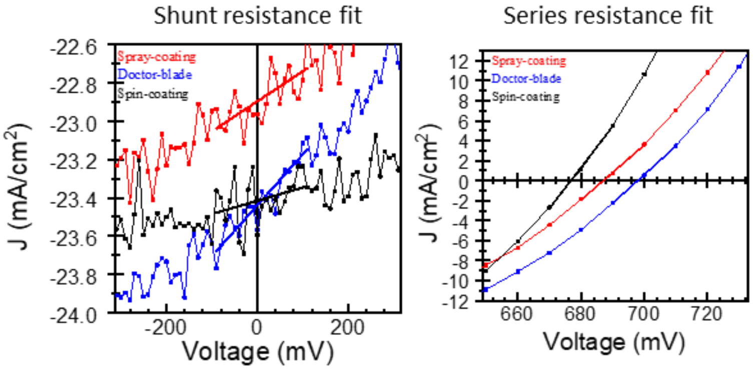

Deposition	Series resistance, R_S (Ω cm²)	Shunt resistance, R_{Sh} (Ω cm²)
Spray-coating	3.6	6.3×10^2
Doctor-blade	3.5	3.8×10^2
Spin-coating	2.4	1.44×10^3

Figure S5. Fittings to obtain shunt and series resistances from J-V curves for devices made with different deposition techniques.

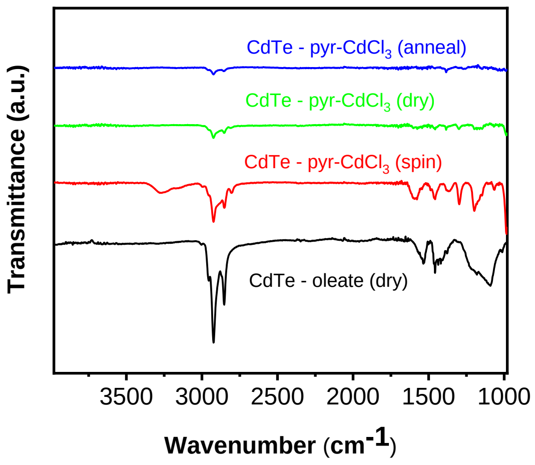

Figure S6. FTIR analysis of (properly-washed) pyr-$CdCl_3$-capped CdTe NCs directly after spin-coating, upon drying at 150 °C and after annealing at 350 °C. The spectrum of oleate-capped CdTe NCs is also shown for reference.

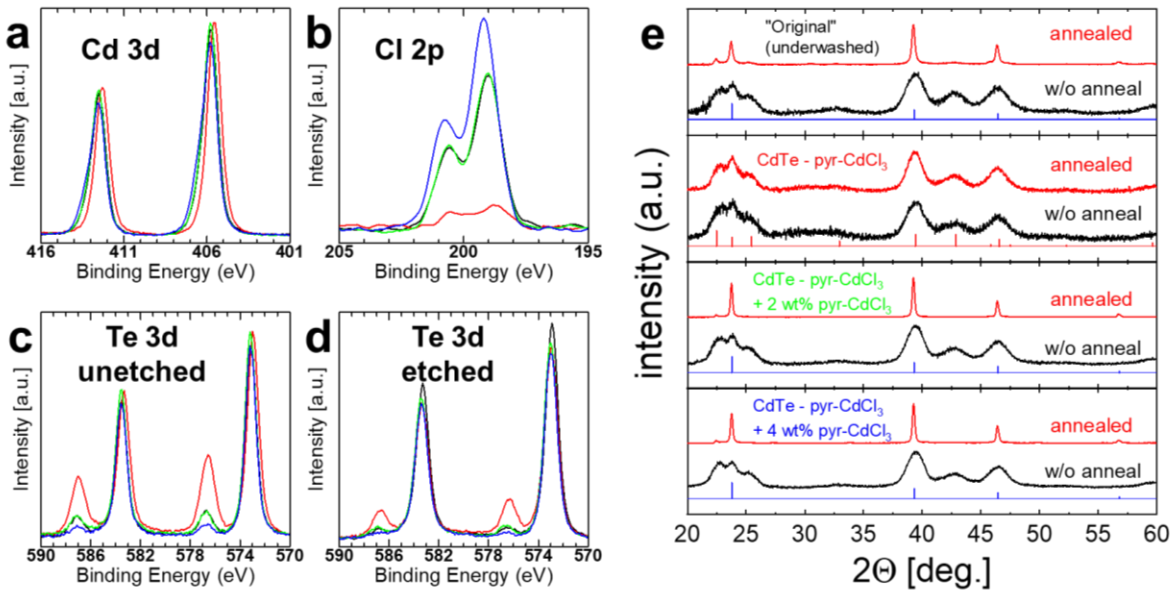

Figure S7. (a-d) XPS spectra comparing sintered single-layer CdTe thin films made from “original”, under-washed pyr-$CdCl_3$-capped NC ink (black), properly washed pyr-$CdCl_3$-capped NC ink with 0 wt% (red), 2 wt% (green), and 4 wt% (blue) pyr-$CdCl_3$ added in (a) Cd 3d, (b) Cl 2p, (c) Te 3d (unetched), and (d) Te 3d (etched) regions. (e) XRD patterns of single-layer CdTe with (red) and without (black) annealing comparing grain growth of the “original”, under-washed ink and the properly washed ink with 0, 2, and 4 wt% pyr-$CdCl_3$ added. The vertical lines show the corresponding X-ray diffractions of bulk CdTe in wurtzite (red) and zinc-blende (blue) phase.

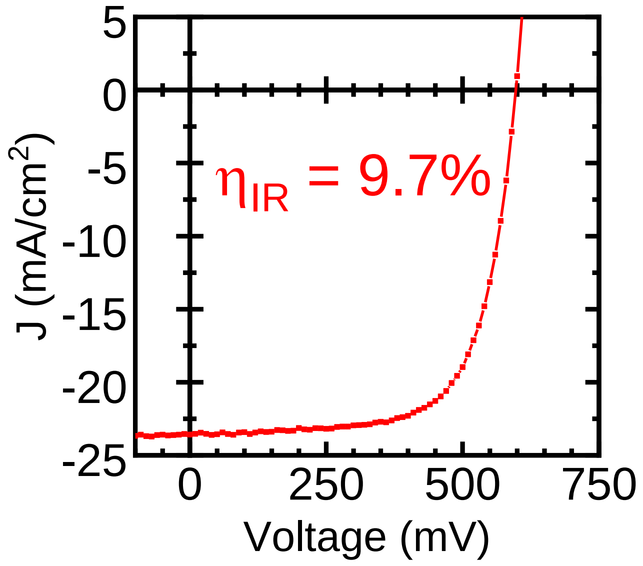

Figure S8. JV curves under AM1.5G illumination for solar cells made with IR lamp used for sintering.

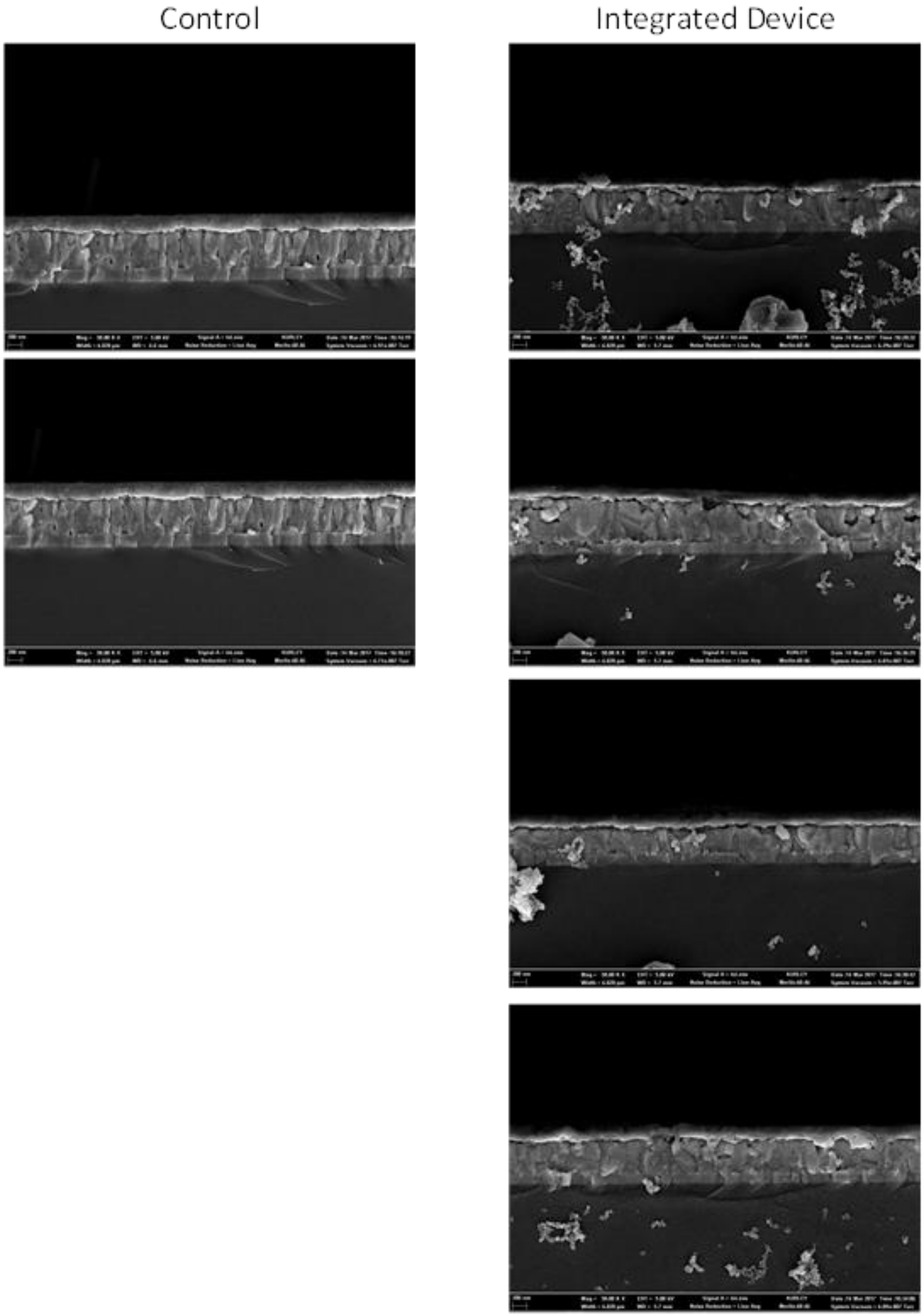

Figure S9. Additional cross-sectional SEM images of devices made or with standard, non-R2R-friendly techniques (left column) or with R2R-friendly techniques (right column).

Scheme S1. Integrated R2R-friendly deposition of sintered CdTe NCs.

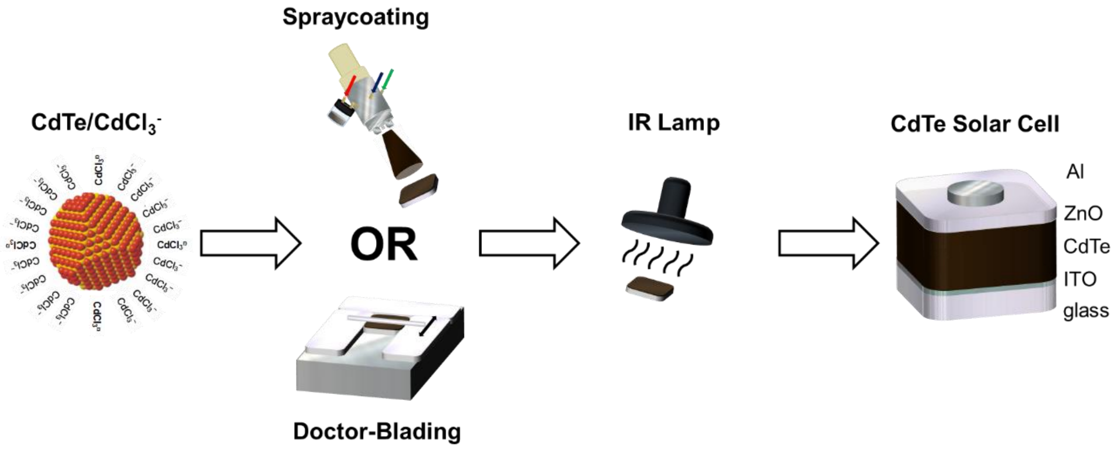

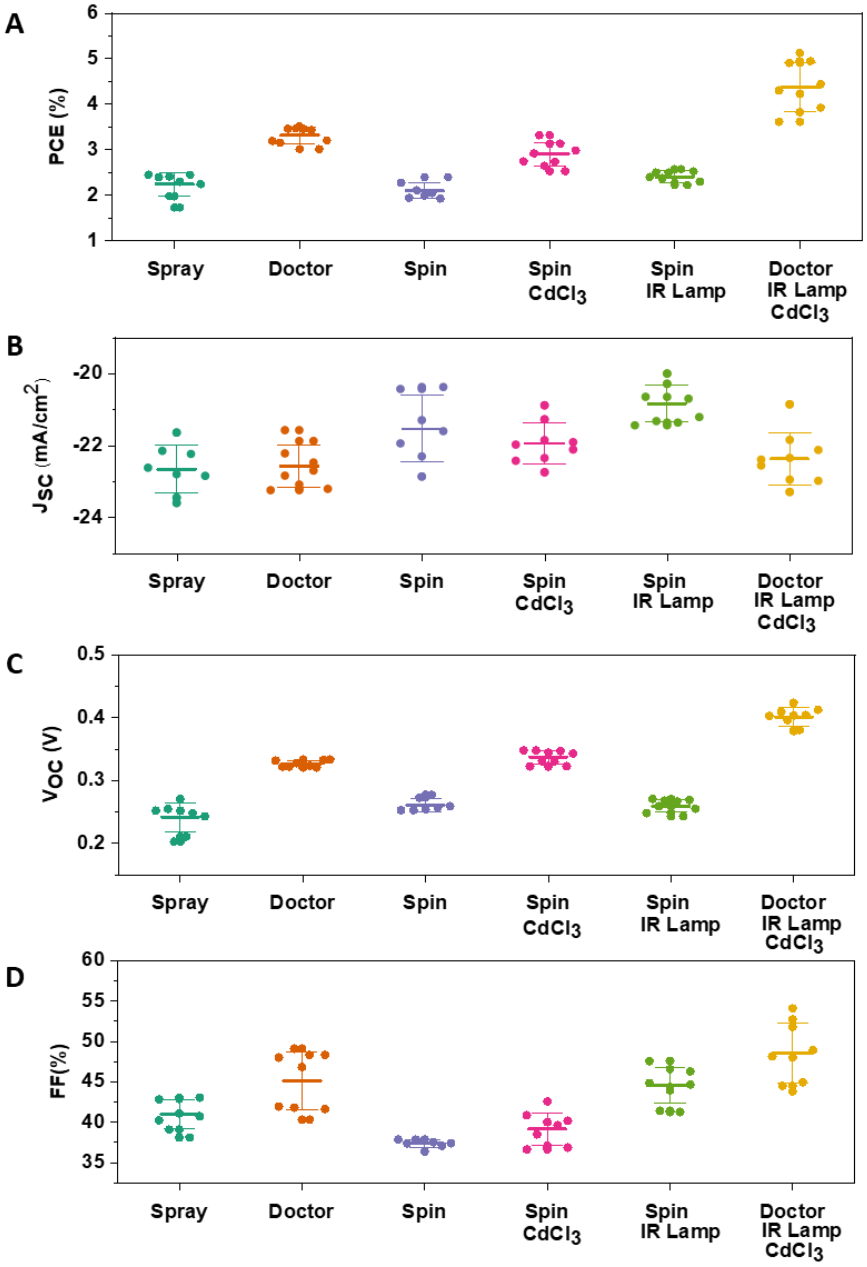

E

Sample	Spray	Doctor	Spin	Spin, $CdCl_3$	Spin, IR lamp	Doctor, IR lamp, $CdCl_3$
# of good pixels	8	9	7	9	9	9
# of bad pixels	1	0	2	0	0	0

Figure S10. Statistics of device performance before current/light soaking. Unless otherwise noted, devices were made using a $CdCl_2$ bath and annealing with a hot plate. (A–D) Power conversion efficiency (A), short-circuit current (B), open-circuit voltage (C) and fill-factor (D) statistics. (E) Table showing the number of good and bad pixels in a 9-pixel device. The bad pixels were not included in the analysis.

Deposition	Series resistance, R_S (Ω cm^2)	Shunt resistance, R_S (Ω cm^2)
CdTe - pyr-$CdCl_3$	467	1.51×10^2
CdTe - pyr-$CdCl_3$ + 2 wt% pyr-$CdCl_3$	3.17	1.14×10^2
CdTe - pyr-$CdCl_3$ + 4 wt% pyr-$CdCl_3$	8.95	1.12×10^1

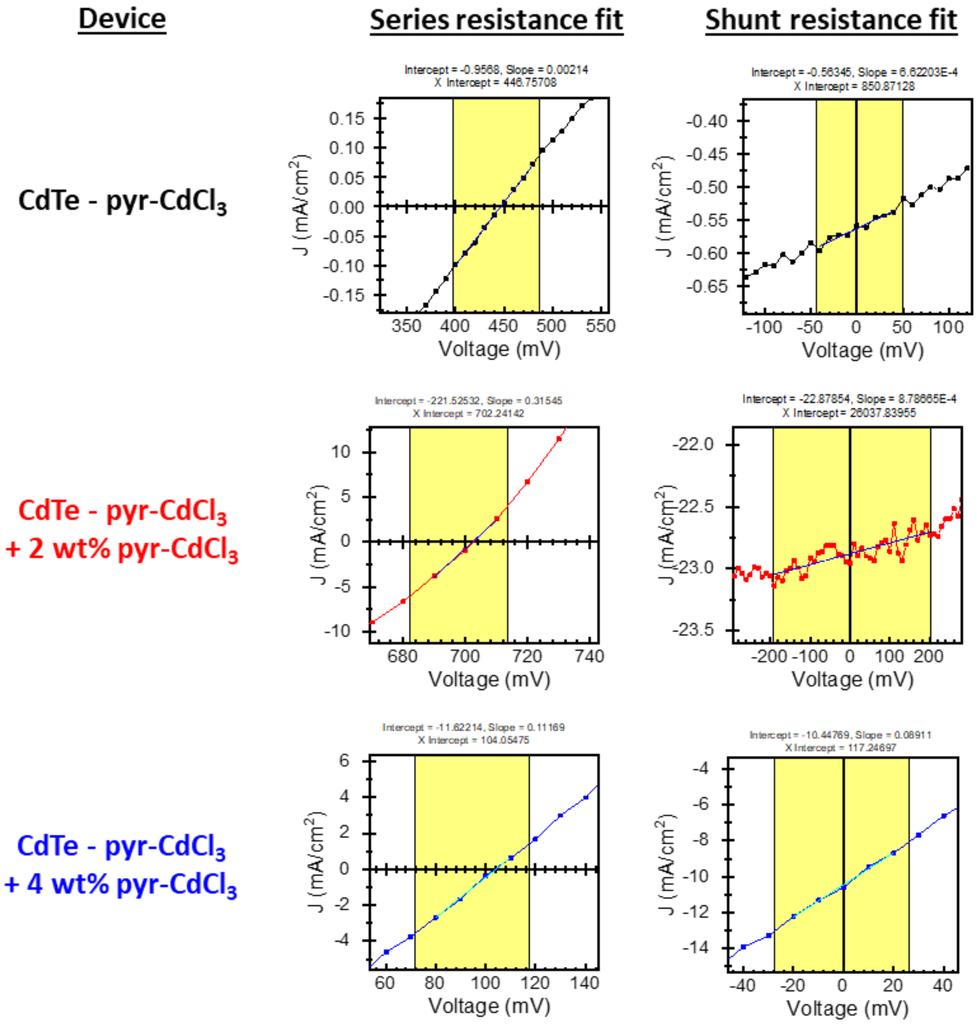

Figure S11. Fittings to obtain shunt and series resistances from J-V curves for devices made with various amounts of $CdCl_2$ added.

Supplementary References

1. MacDonald, B. I.; Gaspera, E. D.; Watkins, S. E.; Mulvaney, P.; Jasieniak, J. J., Enhanced Photovoltaic Performance of Nanocrystalline CdTe/ZnO Solar Cells Using Sol-Gel ZnO and Positive Bias Treatment. *J. Appl. Phys.* **2014,** *115* (18), 184501.
2. Jasieniak, J.; MacDonald, B. I.; Watkins, S. E.; Mulvaney, P., Solution-Processed Sintered Nanocrystal Solar Cells via Layer-by-Layer Assembly. *Nano Lett.* **2011,** *11* (7), 2856-2864.
3. Zhang, H.; Kurley, J. M.; Russell, J. C.; Jang, J.; Talapin, D. V., Solution-Processed, Ultrathin Solar Cells From $CdCl_3^-$ - Capped CdTe Nanocrystals: The Multiple Roles Of $CdCl_3^-$ Ligands. *J. Am. Chem. Soc.* **2016,** *138* (24), 7464-7467.
